# Molecularly imprinted polymers by epitope imprinting: a journey from molecular interactions to the available bioinformatics resources to scout for epitope templates

**DOI:** 10.1007/s00216-021-03409-1

**Published:** 2021-05-20

**Authors:** Laura Pasquardini, Alessandra Maria Bossi

**Affiliations:** 1 Indivenire Srl, via Alla Cascata 56/C, Povo, 38123 Trento, Italy; 2 Department of Biotechnology, University of Verona, Strada Le Grazie 15, 37134 Verona, Italy

**Keywords:** Molecularly imprinted polymers, Protein imprinting, Epitope imprinting, Epitope prediction, Protein database, Bioinformatics

## Abstract

The molecular imprinting of proteins is the process of forming biomimetics with entailed protein-recognition by means of a template-assisted synthesis. Protein-imprinted polymers (pMIPs) have been successfully employed in separations, assays, sensors, and imaging. From a technical point of view, imprinting a protein is both costly, for protein expression and purification, and challenging, for the preservation of the protein’s structural properties. In fact, the imprinting process needs to guarantee the preservation of the same protein three-dimensional conformation that later would be recognized. So far, the captivating idea to imprint just a portion of the protein, i.e., an epitope, instead of the whole, proved successful, offering reduced costs, compatibility with many synthetic conditions (solvents, pH, temperatures), and fine-tuning of the peptide sequence so to target specific physiological and functional conditions of the protein, such as post-translational modifications. Here, protein-protein interactions and the biochemical features of the epitopes are inspected, deriving lessons to prepare more effective pMIPs. Epitopes are categorized in linear or structured, immunogenic or not, located at the protein’s surface or buried in its core and the imprinting strategies are discussed. Moreover, attention is given to freely available online bioinformatics resources that might offer key tools to gain further rationale amid the selection process of suitable epitopes templates.

## Introduction

### Reasons for protein imprinting

Molecularly imprinted polymers (MIPs) are biomimetics prepared by means of a template-assisted synthesis [1, 2]. The specific recognition properties of MIPs are entailed during the polymerization process. The MIP is prepared by the co-polymerization of monomers and crosslinkers in the presence of the target analyte, which acts as a molecular template. At the completion of the polymerization, the template is extracted. The so-formed material retains molecular cavities that are complementary to the template and ready to re-bind it. In fact, MIPs display high affinity and selectivity for their targets, often rivaling those of their natural counterparts, including those of monoclonal antibodies [3, 4]. Additionally, MIPs have the advantage of being polymers, exhibiting tolerance to solvents, to extreme pHs, to temperatures, the possibility of undergoing sterilization [5], robustness, processability, and integrability to electronics.

Relatively to the templates, the imprinting of small molecules is nowadays performed through robust and reliable protocols, while challenges still come when the task is to imprint macromolecules, such as proteins [6], nucleic acids [7], cells, bacteria, and viruses [8–10].

Macromolecules and their higher order assemblies, which lead to the formation of macromolecular complexes, or at a greater size to the formation of organized living machines, such as viruses and cells, have been representing a strategic target to imprint for the last 25 years [6, 11, 12]. Such a long-lasting interest comes from the key roles exerted in living beings by proteins, and by the protein crosstalk, that is central to keep organisms functioning. Diagnosis and prognosis are based on the detection and quantification of certain protein biomarkers; moreover, therapeutics are designed to intervene in protein interaction paths, involved in the onset or in the progression of a pathology. Thus, for the most part, the efforts to imprint proteins have been focusing on solving unmet analytical needs, such as addressing selected biomarkers, for which antibodies are not available or are unstable, etc., both by developing assays [13, 14] and sensors [15, 16]. More recently, the perspective moved to therapy. Protein imprinted polymers (pMIPs) raise expectations for playing more dynamic clinical roles by acting as direct interferents amid cellular interactions, such as playing a role in preventing protein dimerization [17, 18]. The stakes for succeeding with pMIP materials as therapeutics are very high. The partial, yet promising, current level of success arises from the nanosizing of the MIPs that are also referred to as plastic antibodies [3, 4].

From a technical point of view, imprinting a protein is not an easy task, as proteins are polypeptide chains characterized by particular three-dimensional (3D) conformations, or folding, which are essential to keep the protein performing its function (e.g., interaction, catalysis), but that are easily lost including in most of the polymerization conditions in-use for the MIP synthesis [19]. It has been observed that to successfully imprint a protein-binding site, the protein should be stamped when it is in the defined 3D conformation that is lately intended to be recognized and bound by the pMIP. It has been demonstrated that a pMIP prepared towards a folded protein binds the protein when it is in the very same folded state, whereas no binding is observed for the same protein when undergoing conformational transitions that change its 3D shape [20].

Indeed, protein conformation is adapted by nature to be easily disrupted, as this is the physiological mechanism that regulates protein turnover and signaling in cells. Proteins tend to unfold especially when placed in conditions far from their native environments. It results in the protein unfolding when solvated in most of the solvents, as well as in the presence of a high concentration of monomers that modify the pH or the physicochemical characteristics of the solution. The loss of fold is also strongly driven by the temperature of the environment that in free radical polymerization highly increases during the polymerization [21]. The limits to the polymerization conditions posed by the necessity to keep the proteins in their native 3D shapes imply reconsidering protocols for the preparation of MIPs that are state of art. To successfully imprint a protein, the imprinting process should be adapted in terms of solvents, of solubility in the polymerization monomers, buffering conditions, and temperature, so to preserve and maintain the protein folded during the whole MIP synthesis process [22].

Besides the key issue of keeping the protein in its original fold, the other limit to the pMIP technology is the source of the protein template, namely the quantity of available protein at a pure grade and the costs associated to the expression and purification of the proteins. Preparing pMIP can be extremely expensive, hugely restricting the uses of pMIP to the lab scale or to a few proofs of principle applications. Such a scenario de facto is in contradiction with the original claim at the very basis of the MIP concept and with collective expectations for cheap and available tailor-made pMIPs. Additionally, the advancement in pMIP synthesis brought by the immobilization of the template to a solid support sets a general strategy for the preparation of oriented-binding sites in MIPs, as demonstrated already in 2003 by the coupling of amino acids to silica supports [23]. Later, the same idea was successfully translated to the synthesis of MIP nanoparticles [24, 25], further heightening the expectations. Concerning the nanoMIP solid-phase synthesis, surely the in-use protocols require additional improvements, especially in the yields (~10–15%), as the thermodynamics of a free radical polymerization reaction occurring at an interface is undoubtedly unfavorable. Indeed, strategies such as surface-initiated reversible addition fragmentation chain transfer polymerizations have been demonstrated to significantly improve the control of the polymerization [26, 27] and were proven to improve the yields of MIPs [28]. Nevertheless, the idea of coupling a template to a support, hence orienting the template, has empowered the MIP and nanoMIP technologies.

### The lesson from protein-protein interaction: to imprint just an epitope

**Fig. 1:**
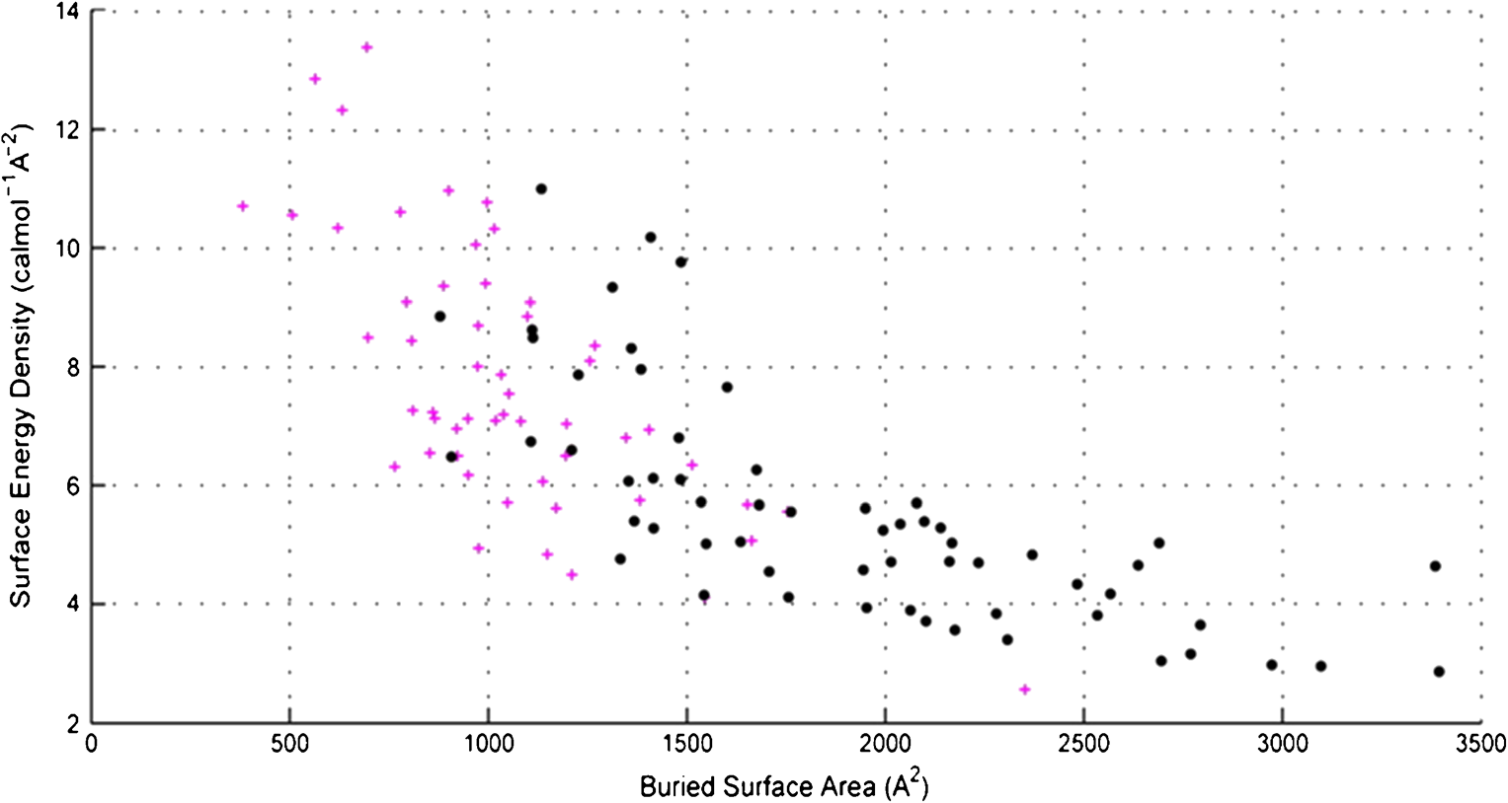
Scatter plot showing the relationship between the free energy change per Å^2^, also indicated as “surface energy density,” and the buried surface area (in Å^2^) of 113 heterodimers. For interactions below 2000 Å^2^, the energy density correlated linearly with the increasing contact area. Above 2000 Å^2^, a plateau is observed. Each point represents a co-crystal structure of a heterodimer: protein-peptide complexes are denoted by magenta crosses and all other protein-protein complexes are black circles. Reprinted with permission from [30]

Taking a closer look at how proteins interact with each other allows learning the secrets of the natural binding sites. The close observation of the macromolecular contact points is a source of inspiration to reproduce similar interactions in the MIPs. Looking at proteins, it appears that the protein-protein interactions occur via defined contact points, which are characterized by interfacial surface areas comprised between 500 and 3500 Å^2^, at least this is valid for the majority of the studied protein pairs [29, 30]. Observations over the contact-involved, or the buried surface area (in Å^2^), for a number of heterodimers (*n* = 113) indicated that if the contact area increases, the free energy change per Å^2^, that is also indicated in terms of “surface energy density” decreased, leading to more stable interactions, as shown in Fig. 1. Surprisingly, it was also noted that contact areas greater than 2000 Å^2^ did not lead to tightened interactions or to significantly higher affinities [30]. Thus, the stability of the pair and affinity constants for binary protein interaction can be inversely but linearly correlated to the surface energy density when the contact areas encompass 800–2000 Å^2^.

Then, for a better understanding of the molecular interactions, it is profitable to analyze each specific contact point, within the interacting area, in a perspective of thermodynamics and energetics. The suggestion, made earlier by Culver and Peppas [31], leads to breaking down the molecular contacts of the interacting area in single energy bond contributions. Surprisingly, very weak types of chemical bonds, such as hydrophobic interactions and pi-pi stacking, are much used by nature to stabilize the pairing, as their total contribution is additive [30]. Moreover, hydrophobic interacting areas have the advantage of sealing off water molecules from the contact, providing a solvent-free environment that represents a microenvironment characterized by low dielectric constants and thus provides a strong driving force to keep the proteins bound by hydrophobic effect. Overall, the analysis of the protein complexes teaches us that protein-protein interactions occur via a defined portion of the protein surface, highlighting the fact that just a selected part of the protein is sufficient to bind or recognize a molecular partner. Additionally, these observations demonstrate that despite the limited surface involved in the recognition, remarkably high strength contacts can be achieved. Indeed, antibodies and receptors bind their targets, showing dissociation constants as low as in the picomolar range, by possessing complementarity just towards a small fragment, i.e., a peptide, of the whole protein. The targeted fragment is called epitope.

**Fig. 2:**
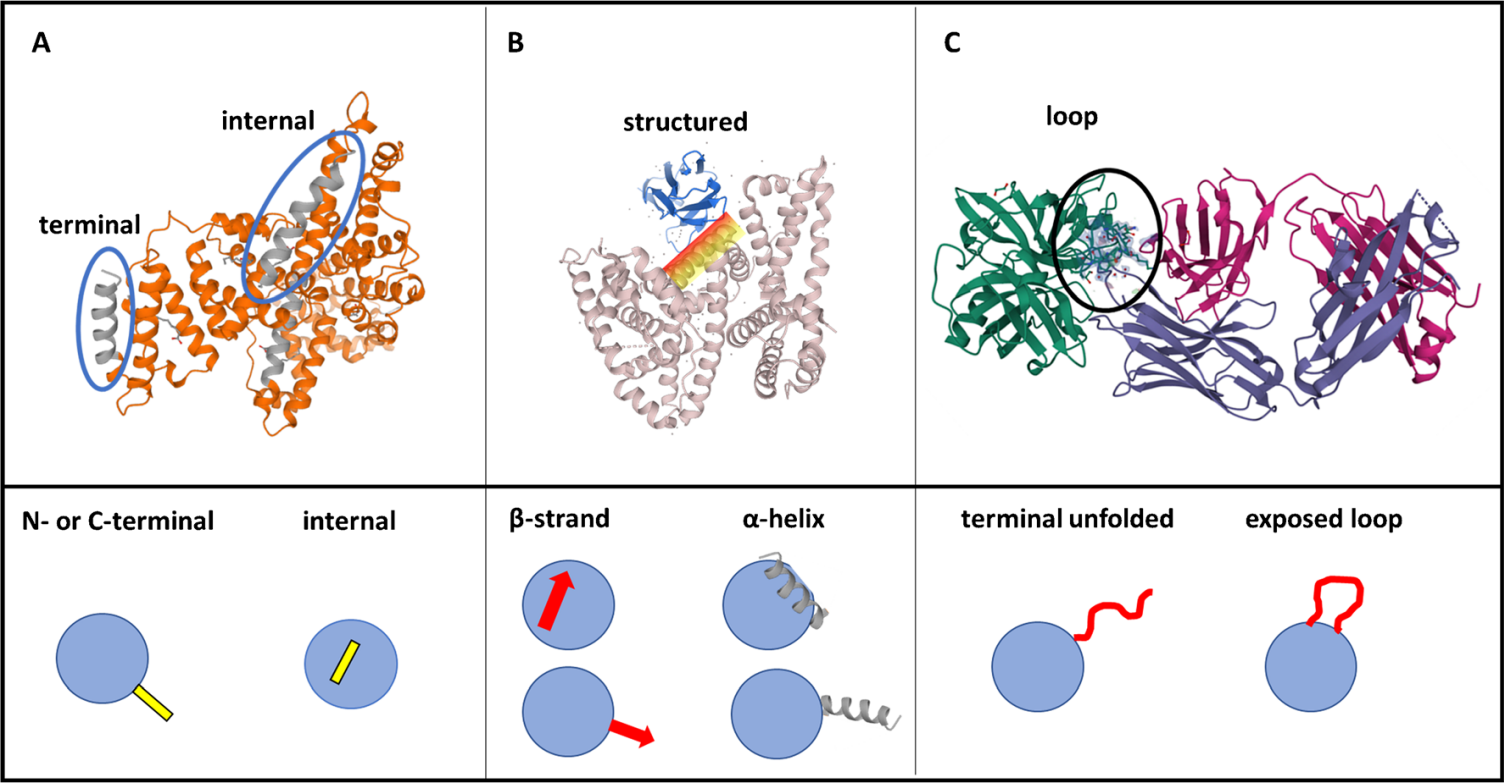
The epitopes arising from a continuous amino acid sequence are the most promising to imprint. Among these we can distinguish: (**A**) epitopes located at the N- or C-terminus of the protein, or epitopes placed within the amino acid sequence, thus internal; (**B**) some epitopes are characterized by a secondary structure, such as a helix or a beta-strand, and can be defined as structured epitopes; (**c**) most of the epitopes lack a properly defined secondary structure and are therefore loose terminal stretches, or flexible loops of the protein, characterized by structural flexibility. Nevertheless, the exposure to the solvent of both the terminal stretches and the loops and their accessibility to binding partners come with a defined orientation and with inherent directional constrains. In the examples, the following structures are shown: (**A**) human serum albumin (HSA); (**B**) co-crystal of HSA with shark IgNAR variable domain (**B**); co-crystal of Fab fragment with human serum kallikrein

Originally, the term epitope was coined by the Danish immunologist Niels Kaj Jerne to define short amino acid sequences exposed at the protein’s surface that were found immunogenic [32]. Epitopes are for the most part stretches of sequential amino acids, located in turns and loops of the protein structure (Fig. 2). Alternatively, a further kind of observed epitope is the conformational, or discontinuous, one. This, instead of being formed by a continuous amino acid sequence, arises from the 3D clustering of few amino acids, or short peptide sequences, belonging to distal portions of the protein sequence, but gathered together in the protein’s final fold. Out of these observations, a smart and promising strategy to imprint a protein-selective binding site on the pMIP is to use an epitope template, instead of the whole protein. This strategy, conceived in 1999 by Rachkov, is called epitope imprinting [33–36].

The epitope that best suits the imprinting process is a continuous sequence of amino acids. In contrast, discontinuous epitopes are not straightforward as templates; thus, to date, these have been avoided for imprinting. The ideal characteristics of an epitope-template are the following: (i) being an oligopeptide, with a typical length of 5–30 amino acids; (ii) the epitope, being a peptide with no precoined fold, withstands a broad range of polymerization conditions, without alteration [36]; and (iii) the epitope is easily prepared by artificial synthesis, significantly cutting the costs associated to biological templates, hence yielding to affordable pMIP materials [36].

Yet, among the continuous epitopes, there is variety of placements and configurations within the parental protein that are briefly summarized in Fig. 2. Such a variety imposes re-adjusting the imprinting process for each kind of epitope, as will be discussed later. In fact, in dependence to its localization within the protein sequence, the epitope can be terminal, such as C- or N-terminal peptides, as reported in several examples [35, 37, 38], or localized within the sequence and therefore internal [39, 40] (Fig. 2A). Moreover, some epitopes are portions of the protein’s sequence characterized by a defined secondary structure, such as a α-helix or a β-strand (Fig. 2B), as in the example of the p32 tumor marker protein [41, 42] and in the case of the α-helix of an HIV protease [43].

Finally, the epitope exposed at the protein’s surface, whether it is located terminally or internally, is characterized by a directional exposure and accessibility both to the solvent and to its possible interacting partners (Fig. 2C); thus, the orientation of the epitope during the imprinting process shall also be carefully considered. Template orientation control can be achieved by exploiting inhibitors, such as benzamidine, that work to directionally immobilize the template, i.e., a serine-protease enzyme via its active site [44], or more generally by coupling the epitope to a solid support in a defined direction, which is a practice that takes advantage of the classical coupling chemistries, such as carbodiimide/succinimide [25], glutaraldehyde [25], and more recently of click chemistry [17]. A further example on how to directionally immobilize an epitope lies in tagging the targeted protein with a common biochemical tag, such as the His-tag [45] or the FLAG tag [46]. Tags are convenient templates that can be immobilized by specific chemistries, such as divalent cations, and chelating affinity supports that produce pMIPs suitable to recapturing His-tagged proteins [45]. Finally, some authors report also the immobilization of non-linear epitopes, as in the example of a cyclic peptide that mimics the amino acid accessibility of the capsid protein gp120 of HIV, followed by its immobilization to a support for imprinting, with the aim of improving the fidelity of the resulting pMIP nanoparticles [47].

At last, when considering which epitopes are worth to imprint, the choice of the peptides goes beyond the structural considerations discussed in Fig. 2. Ideally, the epitope to be imprinted should bear a signature of the parental’s protein and be distinctive of it; this is to assure the selectivity of the formed imprinted binding site. Alternatively, the epitope should be that very portion of the protein that triggers a particular physiological or functional role. In this perspective, the definition of epitope is somehow stretched far from the original immunological and structural conception; instead, the concept of epitope narrows down to include the single amino acid that undergoes a post-translational modification (PTM), or even comprises the PTM itself. This consideration drove the attention towards template epitopes that were signatures of a protein’s phosphorylation [48] and a protein’s glycosylation [49].

### Strategies to imprint an epitope

**Fig. 3:**
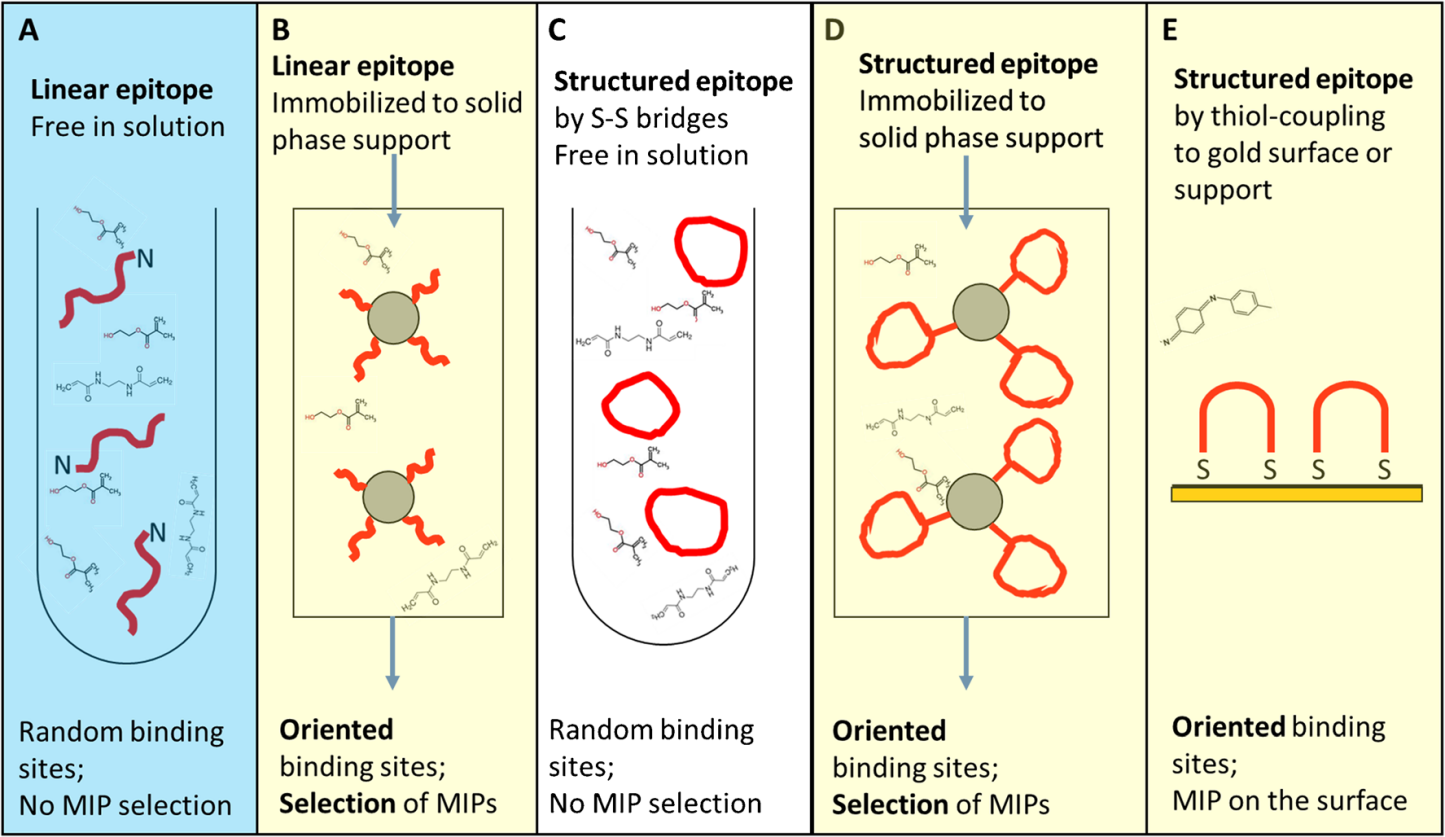
The epitope imprinting strategies. Colors distinguish the different approaches, according to the classification arbitrarily chosen and discussed in the text. Light blue color marks the free in solution synthesis of linear epitopes (**A**). Yellow color marks the onto a solid-support synthesis, in which the epitope is immobilized to a support. The strategy finds application to both linear epitopes (**B**), structured epitopes, being cyclic peptides (**D**), or secondary structures (**D**, not shown), or bridged peptides (**E**); white color marks the free in solution synthesis of structured or conformational epitopes (**C**)

Considering the different kinds of epitopes discussed above, it follows that more than a single imprinting strategy is needed. In fact, three main strategies can be broadly recognized in epitope imprinting, as summarized in Fig. 3. The easiest approach, which is also the one originally conceived, consists of using just the free epitope as a template (Fig. 3A). The template peptide is placed in a solution together with the monomers and the crosslinker and the polymerization is started. It is an “all in solution” synthesis protocol that has the key advantage of being straightforward. As a counterbalance, the in-solution synthesis would not offer control over the stamping process; thus, heterogenous binding sites are expected. Despite this limitation, the free epitope imprinting has been largely utilized, as reported in some examples in Table 1, and the so-formed pMIPs can be still characterized by very high selectivity for the target protein and by dissociation constants in the nanomolar range [39]. The free epitope synthetic approach is also exploited in combination with electro-polymerizable semiconducting monomers, as in an example based on a p-norepinephrine MIP sensing surface imprinted for the recognition of the C- or the N-terminus of the cardiac failure biomarker Troponin I, that reported a K_D_ for the Troponin of 4.4 nM [56], or in the electropolymerization of terthiophenes in the presence of gluten epitopes [57].

**Table 1:** Recent examples of free epitope imprinting

Epitope	Protein	Protein’s role	Characteristics	Results	Reference
Alpha-synuclein peptide 27–39	Alpha-synuclein	Parkinson disease (PD)	-Free template -Electropolymerization	-Epitope alpha-synuclein 27–39 was considerably more effective as an imprinted template than two other epitopes tested -Reasons for differences in imprinting effectiveness remain unclear -Alpha-synuclein detected at fg/mL -Measures in culture medium of human brain organoids generated from normal and idiopathic PD patients	[50]
KDIFHKNNQ 170–178 extracellular domain of HER2	Receptor HER2	Transmembrane protein overexpressed in several cancers	-Dual free templates: epitope and doxorubicin	-Fluorescent dual-template MIP NPs coupled with Si NPs for targeted bioimaging and targeted therapy in HER2-positive breast cancer cells K_D_ and maximal saturation values were 0.77 mL mg^−1^ and 55.2 mg g^−1^ for the MIP IF >5 -Doxorubicin-loaded MIP NPs bind to HER2 and the pH of the microenvironment leads to release of the drug in situ	[51]
P-Gal	Extracellular glycans on red blood cells (RBC)	Recognition of RBC types	-Free epitope imprinting	-Biocompatible MIP nanogels with high binding capacity and binding selectivity towards P-Gal in PBS -MIPs selectively shielded blood group antigen on RBCs -Proof of concept to suppress immunogenicity of blood group antigens for blood transfusion therapy	[52]
71–80 peptide of FN14 with sequence PPAPFRLLWP, Glu-FH and Glu-PPAPFRLLWP and Bleomycin	Human fibroblast growth-factor-inducible 14	Overexpressed in pancreatic cancer	-Addition of glutamic to the 71–80 peptide -Dual template (GluFN14) and bleomycin	- SiNPs@SiO2 onto which polymerized zinc acrylate, 4-Vinylbenzeneboronic acid in the presence of the two templates - Imaging experiments in vitro showed the FH-MIP-NPs specifically targeted BxPC-3 cells -FH-MIP-NP-loaded BLM inhibited the growth of the tumor	[53]
Adenovirus type 5 hexon protein	Adenovirus type 5	Viral infection	-Adenovirus type 5 hexon protein adsorbed to silica beads	-Use of a virus capsid protein as a template -Detect the adenovirus type 5 at 10^3^ IU -Selectivity for Adenovirus type 5 - Virus-imprinted particles bind virus in cell culture supernatants -Permit to circumvent the use of hazardous biologicals during the MIP synthesis	[54]
l-Lysine at the C-terminus	Fc region of immunoglobulin G (IgG)	Immunological response	-Lysine as C term amino acids of Fc portion of IgG was oriented by emulsion	-Selectivity for l-lysine over entantiomer with a factor ca. 3 -The MIP NPs induced oriented immobilization of antibodies - IgG recognition and orientation through l-lysine imprinted nanoparticles	[55]

The step forward in the epitope imprinting was taken when the epitope was immobilized to a solid support. This was earlier proposed by the group of Sellergren [23]. The concept was demonstrated by the immobilization of an epitope template, i.e., the dipeptide Phe-Gly, to silica particles for the generation of hierarchically imprinted polymers. The pores of the silica mold were filled with a mixture of monomers/initiator and polymerized, followed by dissolution of the silica template. The method permitted producing MIPs that have the imprinted binding sites located at their surface, and thus well accessible for rebinding. Moreover, it was demonstrated that the binding sites were selective for the parental heptadecapeptide nociceptin, proving once more the efficacy of the epitope imprinting [58]. Later, the concept of template immobilization was harmonized to that of affinity chromatography thus providing a convenient technology asset for the wide expansion of such imprinting strategy. The idea consists in the use of an epitope-derivatized affinity column to directly perform a solid-phase synthesis of nanoMIPs [24]. As depicted in Fig. 3B, a commercial affinity solid-phase resin is derivatized with the epitope, packed in the column, filled with diluted monomers and crosslinker solutions, and added to the initiator, and the polymerization takes place inside the column. At the end, all the materials not bound to the epitopes, or loosely attached to the affinity resin, are washed away, leaving on the column just the high-affinity nanoMIPs captured onto the immobilized epitopes. High-stringency washings, or a change in temperature, when dealing with thermoresponsive nanoMIPs, permit recovering high-affinity nanoMIPs, characterized by highly homogeneous and directionally oriented binding sites [24, 25]. The approach has been exploited for a number of different epitopes, of which some examples are included in Table 2. For the most part, the directionality has been imparted by choosing a N- or a C-terminus peptide, but directional imprinting can be achieved also by exploiting protein tags of common use in protein expression and purification, such as the His-tag that can form coordination complexes with divalent cations, such as Ni^2+^, that are coordinated to an EDTA moiety immobilized on a Fe3O4@SiO2 particle [45], or the FLAG-tag [46].

**Table 2:** Recent examples of directional imprinting of epitopes

Epitope	Protein	Protein’s role	Characteristics	Results	Reference
CNCKAPETALCTNYCFRN N-terminal α-helix of TGF-β3	Transforming growth factor-β3 TGF-β3	Secreted protein influencing proliferation, differentiation, etc.	-Epitope structured by forming a stable alpha helix via cystine bonds -Orientation by inverse microemulsion polymerization	-Positioning of the epitope at the water/oil interface permitted to imprint the cavities at the particle surface of the MIP NPs - The supplementation of the formed MIP NPs as ligands with abiotic affinity for growth factors in alternative to their biological analogs in tissue regeneration medicine applications -MIP NPs prepared for the target TGF-β3 demonstrate functionality as modulators of cell behavior, on both 2D surfaces and 3D cultures	[59]
DYKDC	DYKDDDDK FLAG tag	Tag for protein purification	-Hierarchically imprinted polymer for the recognition of the FLAG tag -Epitope immobilized into the pores of iodoacetylated silica microbeads through the thiol group of the C-terminus cysteine	-Remarkable selectivity for FLAG peptide recognition -The silane coupling agent play a key role in the imprinting process -The template orientation is crucial for recognition	[46]
EEKKVCQGT N-terminal added of palmitic acid	Epidermal growth factor receptor (EGFR)	Membrane receptor marker of epithelial-derived tumors	-N-terminal epitope added at C-terminal of palmitic acid -Oriented imprinting by reverse microemulsion polymerization in Brij35 in the core quantum dots	- μg/mL MIPs to target EGFR - MIPs accumulate at the tumor site after 20 h -Targeted tumor imaging in vivo	[60]
11-mer CLKAVDHINST, Cys-Ep1 and the 13-mer CAMRLGAEVYHTL, Cys-Ep2	Neuron-specific enolase (NSE)	Cancer biomarker	-Hybrid epitope imprinting by electropolymerization in the presence of two computationally selected and cysteine-modified epitopes of NSE	- AuNPs decorated epitope-mediated hybrid MIPs - 2.5-fold higher sensitivity compared to single epitope imprints - NSE detected in human serum in a concentration range of 25–4000 pg/mL - Imprinting factor, target bound on MIP/control polymer, of 4.2	[61]
N-terminal nonapeptide KLTIESTPF and KLTIESTPF modified with fructose attached to the lysine	Carcino-embryonic antigen (CEA)	Cancer and inflammation biomarker when abnormally glycosylated	-Double recognition towards a target glycoprotein by two different types of MIPs, using epitope-imprinted - One is gold nanoparticles (AuNPs)-coated slide as capturing substrate to recognize the peptide epitope -Second glycans-imprinted Raman-active silver nanoparticles as labeling nanotags	-Double recognition strategy specific for the detection of glycoproteins -Improved specificity as compared with single recognition-based assays - Ultrahigh sensitivity, yielding a limit of detection of 5.56 × 10^−14^ M -Allowed the quantitation of carcinoembryonic antigen (CEA) in human serum	[62]
His-tag	His-tag	Tag for protein purification	-His tag epitope on the surface of the magnetic nanoparticles	-His-tag-epitope imprinted magnetic nanoparticles - Specific recognition and separation of thyroid peroxidase antigens	[63]

A further kind of directional immobilization relies on exploiting boronic acid moieties or its derivatives, as these would provide anchoring functions to fix the sugar moieties of glycoproteins, through reversible covalent bonds. The subsequently polymerized MIP would possess oriented sugar-binding sites. The immobilization of a glyco-epitope was a concept extensively developed by the group of Liu [27, 64]. This permitted entailing very selective binding sites in the formed MIPs. As an example, a plasmonic immunoassay based on the glycol-oriented MIP allowed achieving ultralow detection limits (1.5 × 10^−14^ M) for the clinical marker α-fetoprotein [65]. An alternative method for the orientation of the glycoprotein selective binding site that was proposed with the aim of targeting the glycosaminoglycan hyaluronic acid was to conjugate an azidopropyl-modified glucuronic acid to propargylated glass beads and then make use of the solid-phase synthesis; the resulting nanoMIPs were suitable for bioimaging of cell glycans with a K_D_ of 800 nM [66].

A similar concept, though relying on non-covalent interactions, was proposed for the preparation of phospho-selective MIPs that were intended to bind and enrich phosphorylated proteins, as needed in phosphoproteome studies [67]. The chosen epitopes, which were either just the phosphorylated amino acids (Fmoc-pSer; Fmoc-pTyr) or in some cases the phosphorylated peptides, were immobilized by host-guest chemistry thanks to a urea-based functional monomer, in a 2:1 stoichiometric ratio to the template [48, 68]. The synthesized MIPs resulted in competitive respect to the state of art methods for phosphoproteome enrichment. In particular, pTyr-MIPs provided a superior enrichment of the pTyr-modified proteome, when compared to state of art phosphoenrichment methods [69]. In summary, the immobilization of the epitope to a support implies both to choose a convenient coupling chemistry or host-guest chemistry and to burden the MIP synthetic protocol of coupling steps, but in turn it offers the key advantage of choosing the orientation of the epitope, thus directional imprinted binding sites are obtained.

Lately, protein imprinting met a fertile and new area of expansion, foreseen in the concept of pMIPs for biological applications. This led researchers to face and find answers to the issue of achieving superior levels of recognition, adequate to properly mimic natural receptors and antibodies in physiological conditions. These natural environments are seldom reproduced in the analytical conditions. The physiological environments pose thermodynamic constraints to the protein to partner with an interactor, including to a pMIP. In the cell or in the extracellular fluids, proteins are extraordinarily crowded. As an example, the cytoplasm of a cell contains proteins at about 100 mg/mL [70], whereas the total macromolecular concentration, including proteins, lipids, nucleic acids, and sugars, can be as high as 400 mg/mL [71]. Considering the sole protein content and considering it represented by just ideal mean protein having a molecular weight of 50,000 g/mol, it results in the protein concentration in the cytosol being around 2 mM. It can be estimated that in the 2 mM concentration the proteins are distanced from each other by about 6 nm, which is a very short distance, when considering that an average protein hydrodynamic size is 5–8 nm [72]. Moreover, when considering globally all the components present in the cytosol, the crowding effect results are far more significant and the space between a protein and another molecule is reduced to less than a 1 nm. Therefore, when tackling protein-protein interactions with MIPs in a physiological contest, a next level of recognition is invoked, which implies considering that the epitopes are sometimes structured and thus exposing the sequence for recognition both with a direction and with a particular stereochemical organization. We can therefore arbitrarily define a third epitope-imprinting strategy that relies on the attempt to imprint conformation, by means of conformational epitopes specifically designed for the imprinting. In the case, a precursor linear epitope is artificially synthesized but added for conformational constraints, so that its final spatial arrangement mimics the native conformational epitope (Fig. 3C–E). One of the first examples of conformational epitope consisted in the imprinting of the peptide apamin that is designed with disulfide links so to form a stable α-helix and that was used to mimic the extracellular, structured N-terminal part of a protein (p32) associated with tumors [41]. The combination of a structured epitope and surface molecular imprinting produced a nanocarrier that recognized p32-positive tumors in vivo. Later, the idea of the helix epitope was again exploited, but in this case the target was a portion of a HIV protease (PR) that presents a structural interplay between conformation and dimerization. For this, the epitope-peptide (I85-G94) linear sequence was forced to coil in a helix by the addition of a trifluoroethanol (TFE) that is a solvent known for its helix-inducing effect. The results demonstrated remarkable affinity, with K_D_ in the pM range for the binding of the pMIPs to the helical epitope, or to the whole parental protein [43]. An original and distinguished example of structured epitope cleverly copied those proteins that are naturally conformationally disorganized, but that—upon binding to a partner—undergo disordered-to-ordered transition. Among these, the N-terminal transactivation domain of p53 is reported to be originally flexible in solution. Upon encountering the target, such epitope can form a right-handed α-helical structure. A 50% TFE/water solution was thus used for the molecular imprinting reaction in solution, in which the epitope peptide was stamped in the form of α-helix. The results demonstrated that linear peptides in the presence of the helix-imprinted binding sites turned into helixes [73].

A different method to prepare conformational epitopes is to flank a linear peptide-epitope by two cysteines, one to each end and to induce the formation of a cyclic peptide [74]. The cyclic peptide is assumed as a mimic of a loop-shaped conformation. Indeed, a 9-mer cycle representing the apical loop of the biomarker of iron uptake, hepcidin-25, was used as template. The cycle-imprinted binding sites showed the privilege of recognition for the cyclic 9-mer or for the parental protein, with respect to the linear peptide [74]. Moreover, it was observed that the cyclic stamps promoted the catalysis of the linear sequence to the cyclic form, affirming the point that certain fold-imprinted stamps tend to induce that particular fold. As an independent confirmation, in another work, MIPs stamped for the recognition of a cyclic peptide were indeed exploited for catalytic purposes, providing a kinetically favorable environment for the cyclization of linear peptides [75]. Yet, the structural and cyclic peptides can be used either in a free-in-solution imprinting approach (Fig. 3C), or can be immobilized to a support [47] (Fig. 3D). Classical coupling chemistry was used in the case of the CGSWSNKSC epitope, which contains the conserved 3S motif of the envelope glycoprotein 41 of HIV but was added to two cysteine residues to the two sides, so to generate a cyclic structure, that was immobilized on (3-aminopropyl)triethoxysilane (APTES)-glass beads for the imprinting. In this case, the binding isotherms suggested IC_50_ of 18.7 nM for the cyclic template, in contrast to IC_50_ of 69 nM for the linear peptide and IC_50_ of 1500 nM for a larger and mismatching cycle, and of about 1 order of magnitude greater (167 nM) when the competitor is a cycle including some 3S key hydrophobic residues [47]. The results proved the key role of the conformation in fitting to the binding site and shed some light in the important contribution of hydrophobicity on the recognition process.

At last, a fairly recent strategy proposed both to structure the epitope and simultaneously to anchor it to a solid support, so to provide the correct orientation, was based on three ingredients: the addition of one cysteine at each end of the epitope, a gold support, being this a flat surface, but not solely, and the exploitation of thiol-coupling chemistry [76]. The concept is depicted in Fig. 3E. The attractiveness of the strategy lies in the use of the thiol chemistry for the peptide immobilization, which offers robust protocols for producing ordered self-assembled monolayers of peptides. When peptides are anchored to the surface by both ends, they are spatially arranged in the form of bridges that ultimately mimic protein conformational loops and turns. The bridged epitope SAM is the substrate for the imprinting. In an example of electropolymerization of p-scopoletin, a well-controlled and thin MIP film was produced. The recognition results for these highly controlled MIP films are remarkable, indicating a K_D_ of 2.6 × 10^−11^ M and high selectivity towards the target parental protein. The approach is promising and opens future directions.

### The choice of the epitope template

The epitope-synthetic strategies have been providing a number of approaches for preparing the pMIPs; however, these represent just half of the key towards a successful pMIP. To complete the discussion, attention needs to be given to the issue of the selection of the best epitope template, in a given designed application and in a given protein. How to select the epitope is a question that requires rational answers.

Very often, the epitope is chosen for its patho-physiological role and for the impact that that very piece of protein has on the phenomenon we want to observe or measure. Examples of such epitopes are post-translational modification, such as phosphorylation [48, 77–80] or glycosylation [49, 71]. However, the opportunity to identify an imprintable epitope should be generalized to all proteins. In this perspective, there are both attempts of experimental kind and attempts that rely on in silico resources. A promising experimental approach has been reported recently and consists of identifying surface-accessible peptides from the target protein by an approach that starts from the immobilization of the target protein to a solid support [81]. To prove the principle, human hemoglobin (Hb) was immobilized on silica nanoparticles and then fragmented by tryptic digestion. Digested portions of the proteins were washed away. Exposed peptides were left on the support. The particle-supported peptides were then used as templates to synthesize the Hb-selective MIPs. The silica support and the peptides were then removed and the MIPs were tested in equilibrium binding experiments to evaluate their protein separation performance, showing the ability to rebind Hb with high selectivity, including separation of different variants of Hb from crude cell extracts. Whether the solid support, that is in first instance protecting the surface-exposed peptides from cleavage, can hamper to some degree the stamping of the binding sites, this should still be investigated. Nevertheless, the strategy appears as a fast and facile experimental workflow to imprint surface epitopes.

Alternatively, bioinformatics and proteomics databases and analysis software could provide robust in silico platforms for the analysis of protein structures and the final identification of epitopes. Strategies towards a rational evaluation of both the accessibility to solvents and the stability of the chosen epitope have been recently studied with the aim of finding rational routes for the effective formation of high-affinity binding sites [82]. In particular, authors proposed the use of molecular dynamic calculations to define the in-water and in-buffer stability of a panel of chosen epitopes, which were surface-exposed peptides of the protein neuron-specific enolase (NSP). The results demonstrated the key role of the stability prediction of an epitope over the success of its imprinting. Particularly positive appeared the integration of the conformational stability prediction together with a controlled stamping method that produces surface imprints and yields to thin MIP layers [83]. Indeed, the combination of the rational selection and thin MIP synthesis enabled the production of imprinted binding sites for NSP characterized by an apparent K_D_ of 5.3 10^−11^ M. These results suggest the key effect of in silico methods to investigate the structure of the epitope, hence to make a more rational selection of the epitope template.

### Bioinformatics for the choice of the epitope template

Along this line, a general solution to search for many structural or functional characteristics of the epitopes, whether they are functional roles in patho-physiological processes or possess immunogenicity, can be found in bioinformatics. Bioinformatics tools have been facing enormous progress over the last years. In particular, protein databases and the associated interrogating software are in the spotlight for their role in unraveling the global functional behavior of proteins, hence permitting the study of the functional proteomic. These bioinformatics resources are becoming the key tools for ultimately deciphering the whole network of molecular interactions that yield to the functioning of a biological system, driving finally to map the whole systems biology [84–86].

**Fig. 4:**
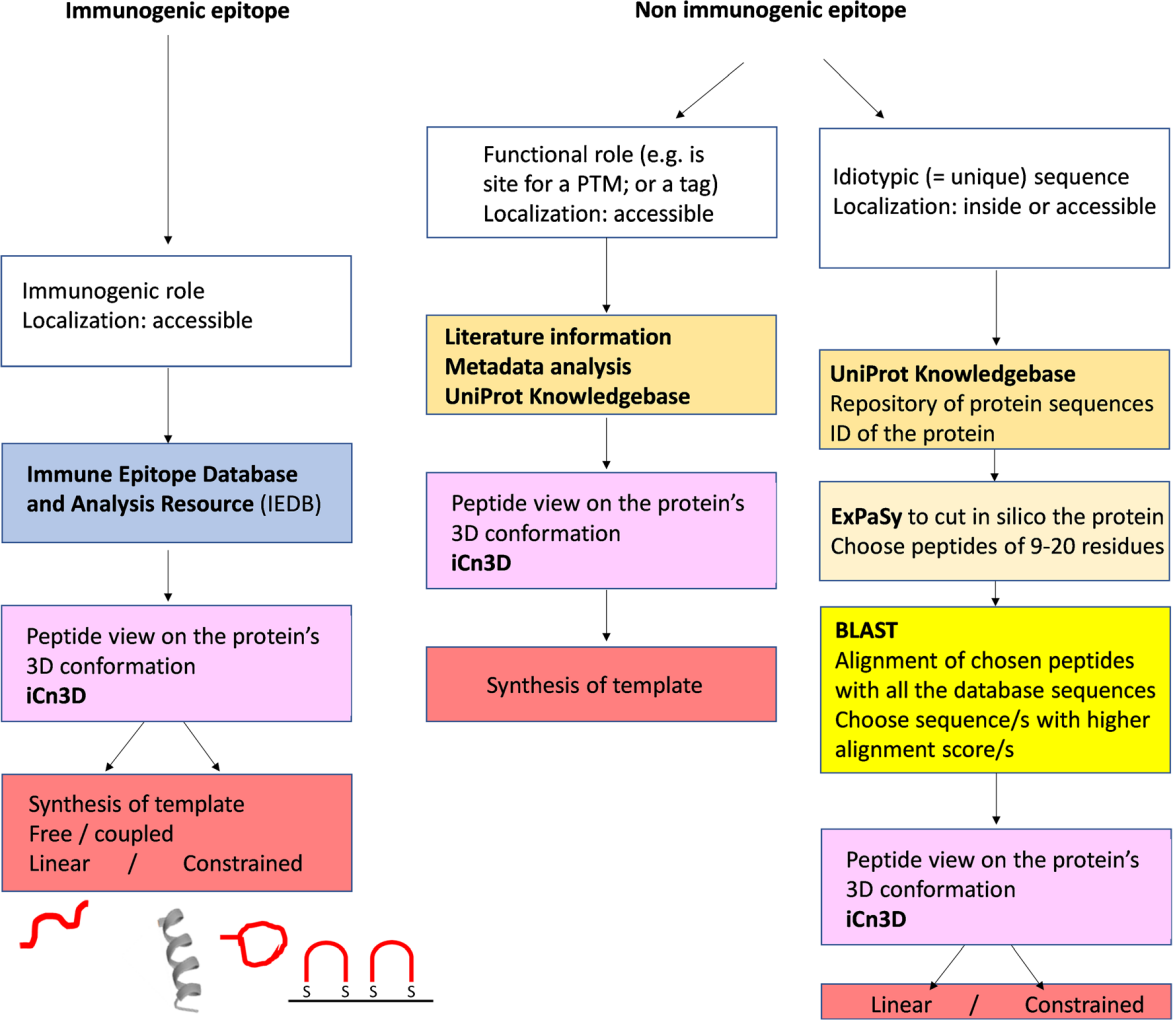
Starting from the type of epitope envisaged for the imprinting, a summary of the workflows is proposed, together with the bioinformatic resources available and with the main aim to use them. Each search funnels down to the definition of an epitope-template sequence, that can be used as free peptide, or coupled to a support and that can be either an unconstrained peptide or built with constraints to impart conformation or directional exposure to the residues composing it

Among the protein repositories and the bioinformatics tools developed for functional studies, some can offer great help in choosing for the epitope to imprint. Therefore, we propose to guide the reader to a better and more global view of these bioinformatics resources. A summary on how to use the bioinformatic resources available to define the best epitope for the imprinting is proposed in Fig. 4. It should be remarked that most of these resources are freely online available, are constantly updated, and are curated protein repositories; hence, these become recommended platforms to get access to and to interrogate when looking for the best epitope template to prepare a pMIP. The bioinformatic tools can be broadly divided in three categories: (1) databases addressed at the selection of those epitopes that are reported as immunogenic; (2) protein databases; (3) structural databases.

### Immunogenic epitopes

If the template will be an immunogenic epitope, there are free bioinformatics tools and curated repositories that store data relevant to immune reactions and specific pathogens. Among these, the Immune Epitope Database and Analysis Resource (IEDB) is a freely available resource that contains an extensive collection of experimentally measured immune epitopes and a suite of tools for predicting and analyzing epitopes [87]. The database is funded by the National Institute of Allergy and Infectious Diseases (NIAID). Data are curated from peer-reviewed scientific literature and from data submitted by researchers. As of January 2021, over 21,600 references have been curated, and the database contains over 787,000 peptide epitopes and over 1,880,000 B cell, T cell, MHC binding, and MHC ligand elution assays (positive and negative). The database is available at the address http://www.iedb.org/. It offers choices between epitopes of sequential or discontinuous sequences; the organism in which the epitope shall be present; the disease associated to the epitope; the type of assay through which the epitope has been identified, allowing performing more and more stringent searches. Filled with the specific input information, the result of the search is a list of epitopes, each associated to the sequence of the peptide epitope and linked to patho-physiological information that help to make the selection.

### Linear epitopes

When there is no need to target an epitope with a specific immunogenic role, the choice of a general linear, or sequential, epitope template is suggested. Moreover, if there is no need to target specific sequences, such as phosphorylation sites, N or C terminus, it is suggested to search for a linear sequence of the protein that is proper and idiotypic (i.e., unique) for that very protein. This goal can be simply and effectively achieved through a search in a protein-sequence repository. These repositories are intended for free consultation, are web-curated, and are apt to the storage of protein sequences. Moreover, they provide software tools for the in silico digestion of proteins and sequence-alignment tools for sequence comparison [88]. Among the available protein repositories, the UniProt Knowledgebase (UniProtKB) [89] is one of the most widely known and used. It is defined as the central hub for the collection of protein sequences and functional information on proteins, with accurate, consistent, and rich annotation [89]. Each UniProtKB entry contains the amino acid sequence, protein name or description, taxonomic data and citation information, the biological ontologies, classifications and cross-references, and indications of the quality of annotation in the form of evidence attribution of experimental and computational data.

UniProtKB allows obtaining the target protein sequence, which is the starting point in the epitope selection process. Then, the protein sequence should be cut into peptides in silico. This can be performed by means of a tool provided by the Expert Protein Analysis System (ExPASy) portal that has software for sequences manipulation, e.g., the in silico enzymatic cleavage of the protein sequences [90]. As a result, the protein will be cut into its constituent peptides. Among the generated list of peptides, there will be some that are too short for being uniquely associated to the parental protein; hence, these are normally discharged, as explained in [91]. Instead, there will be peptides that have a significant length (9–20 amino acids), making their sequences statistically unique. Unique epitopes are called idiotypic. Finally, there will be unique peptides that are longer than 20 amino acids, but these, given the length, might coil up in secondary structures that are not necessarily similar to the natural conformations within the parental protein: these are again to discharge. At the end of the process, it is expected to have a list of possible peptide candidates with length of 9–20 amino acids. Next, the choice of the unique, idiotypic peptide, which is the one that will enable, once imprinted, the univocal binding to the pMIP of the desired target protein, proceeds through the use of in silico tools to align one by one each candidate peptide to the whole protein sequences stored in the protein databank. Among the tools for sequence alignments, there is the Basic Local Alignment Search Tool (BLAST) [92], provided by the National Center for Biotechnology Information. BLAST finds regions of local similarity between sequences and can be used to infer functional and evolutionary relationships between sequences. The sequence similarities are expressed in number, so that the comparison between the scores of each peptide alignment enables to easily rank the similarity [93]. A flowchart of the selection process is reported in Fig. 4.

The result of the process yields to the identification of an epitope peptide that has the maximal probability to be unique. Yet, it should be noted that such an approach permits the identification of unique peptide sequences that can be located either at the protein surface or can be buried inside the protein’s core. Without further refinement, such as screening the idiotypic peptides against structural protein databases, the present search permits designing pMIPs apt for the enrichment of the target peptide from digested proteins. It can be used for shotgun proteomics and for quali-quantitative purposes towards defined protein candidates, always tested in a digested form, as shown in few examples [40, 57, 91].

### Structured epitopes

Bioinformatics helps also when in the search for structural epitopes. Often, epitopes are localized at the surface of a protein and are forming the so-called loops and turns, which are short peptides protruding to the solvent. These peptides are accessible to possible binding partners, including to pMIPs. Despite these epitopes are flexible and do not have a proper conformation, there are significant structural constraints, arising from the insertion of the N- and C-term ends of the peptide into the more tightly packed secondary structures of the protein. In this case, it is convenient to gain structural information over the epitope from tools that allow to define the epitope’s localization in the general protein 3D structure and define their orientation in the structure. Indeed, these information are crucial to choose whether the epitope template should be let in its linear undefined form, or whether it is best to prepare constrained and directional peptides mimicking the natural peptide exposure and orientation in the folded protein. The insight into the structure of the protein can be achieved thanks to structural tools that enable the three-dimensional (3D) vision of the protein. There are several sofware available, such as PyMol or RasMol. In general, interactive 3D structure viewers, such as iCn3D (“I see in 3D”), are software that enable localizing epitopes in the protein structure (Fig. 4). In particular, iCn3D is a WebGL-based viewer for interactive viewing of three-dimensional macromolecular structures and chemicals on the Web that does not require installing a separate application. It can be accessed from the “molecular graphic” that appears on the structure summary page for any record in the Molecular Modeling Database (MMDB) [94]. The topological information related to the protein are linked out to databases that store experimental evidence over the protein-protein interaction areas, links to literature metadata, and predicted information gathered by any available protein-protein interaction databases (see for example: http://string-db.org; https://www.ebi.ac.uk/intact/), providing a panel of criteria for the selection of a one final epitope sequence. The use of oriented and structural epitopes has been gaining increasing attention over these last years. Structural epitopes are envisaged as a means to improve the selectivity of the imprinted binding site towards protein conformations and in practice structured peptides can be obtained by use of cyclic peptide epitopes [47, 74], synthesized with cysteines at both ends, or by the coupling of the ending to a support, thus forming bridged epitopes [76]. Indeed, the strategy offers potentials that are still to be explored by imprinters.

## Conclusions

We witness to significant improvements in the protocols for forming protein imprints, with the aim of achieving high fidelity in the recognition and with the final goal to get pMIPs suitable to satisfy the requirements posed by the intended biomedical applications. In addition to the technological advancements, the preparation of pMIPs is expected to take great benefits from the available bioinformatics protein-addressed resources. The crosstalk between polymer chemists, synthetic chemists devoted to the design of specific monomers for defined interactions, such as phospho-recognition [95, 96], protein structurists, and bioinformatics, is foreseen as the key to enable the formation of selective pMIP binding sites that will perform the unique recognition towards the target protein, also in extremely harsh biological conditions, such as the high degree complexity of the protein landscape in vivo.
